# Burdens of stomach and esophageal cancer from 1990 to 2019 and projection to 2030 in China: Findings from the 2019 Global Burden of Disease Study

**DOI:** 10.7189/jogh.14.04025

**Published:** 2024-01-05

**Authors:** Qianwei Jiang, Yiyang Shu, Zhongyi Jiang, Yanqiang Zhang, Siwei Pan, Weihao Jiang, Jinxiao Liang, Xiangdong Cheng, Zhiyuan Xu

**Affiliations:** 1 Department of Gastric Surgery, Zhejiang Cancer Hospital, Hangzhou, Zhejiang, China; 2 Key Laboratory of Prevention, Diagnosis and Therapy of Upper Gastrointestinal Cancer of Zhejiang Province, Hangzhou, China; 3 Department of Thoracic Surgery, Zhejiang Cancer Hospital, Hangzhou, Zhejiang, China; 4 Department of Ophthalmology, Tongji Hospital, School of Medicine, Tongji University, Shanghai, China; 5 Department of General Surgery, Shanghai General Hospital, Shanghai Jiao Tong University School of Medicine, Shanghai, China

## Abstract

**Background:**

Stomach and esophageal cancer exhibit high morbidity and mortality rate in China, resulting in substantial disease burdens. It is imperative to identify the temporal trends of stomach and esophageal cancer from 1990 to 2019 and project future trends until 2030, which can provide valuable information for planning effective management and prevention strategies.

**Methods:**

We collected and analysed data from the Global Burden of Disease (GBD) between 1990 and 2019, including incidence, mortality, disability-adjusted life years (DALYs), age-standardised incidence rate (ASIR), mortality rate (ASMR) and DALYs rate. We also calculated and reported the proportion of mortality and DALYs attributable to risk factors by sex in China and different regions. The Bayesian age-period-cohort model was applied to project future trends until 2030.

**Results:**

The new cases, deaths and DALYs of stomach and esophageal cancer increased from 1990 to 2019. However, the ASIR, ASMR and age-standardised DALYs rates for stomach and esophageal cancer all decreased during the same period. These changes may be related to risks, such as smoking and diet. Furthermore, we utilised the projection model to estimate that the ASIR and ASMR of stomach and esophageal cancer among females will likely follow steady downward trends, while the ASMR of stomach cancer among males is expected to exhibit a significant decline. However, the ASIR of stomach and esophageal cancer and the ASMR of esophageal cancer among males are projected to display slight upward trends until 2030.

**Conclusions:**

The analysis of stomach and esophageal cancer trends in China from 1990 to 2030 reveals a general decline. However, it is crucial to acknowledge the persistent high burden of both cancers in the country. Adopting healthy lifestyle practices, including the reduction of tobacco and alcohol intake, avoidance of moldy foods and increased consumption of fresh fruits and vegetables can contribute to mitigating the risk of stomach and esophageal cancer. Significantly, the formulation and implementation of well-founded and efficacious public health policies are imperative for alleviating the disease burden in China.

Stomach and esophageal cancer are two major types of the most prevalent cancers, with both their morbidity and mortality ranking in the top 10 among all cancers [1,2]. Globally, stomach cancer has been reported to rank fifth in incidence and fourth in mortality [3,4]. Esophageal cancer ranks seventh and sixth worldwide, respectively [5,6]. Notably, China is the most common country for esophageal cancer, which accounts for approximately 50% of global incidence and death cases annually [7]. According to cancer statistics in China for 2022, esophageal cancer has the sixth-highest national incidence rate and the fourth-highest national mortality rate [8–10]. Stomach cancer ranks third highest in both new cases and deaths in China [10,11]. Moreover, stomach and esophageal cancer predominantly occur in males, with incidence rates approximately 2-fold higher among males than females [12].

These cancers can be divided into different types, each with distinct morbidity and mortality rates [13,14]. Stomach cancer is generally classified into two primary tumor sites: cardiac and noncardiac [15,16]. The incidence rate of cardia stomach cancer has been alarmingly increasing in recent years, potentially due to its main risk factors, including obesity and gastroesophageal reflux disease [17]. However, the occurrence of noncardiac stomach cancer has been decreasing, mainly attributable to the decline in *Helicobacter pylori* infection [18]. Additionally, esophageal cancer comprises two common histologic subtypes: squamous cell carcinoma and adenocarcinoma [19]. While North America and Northern Europe report more cases of esophageal adenocarcinoma than esophageal squamous cell carcinoma, China has a higher incidence of esophageal squamous cell carcinoma annually [5]. Rapid advancements in diagnosis and treatment have improved overall death rates for both cancers, particularly for early-stage cancer [20]. However, many patients are often diagnosed with advanced cancer and cannot benefit from current treatments, leading to substantial disease burdens [21].

The Global Burden of Diseases, Injuries, and Risk Factors Study (GBD) 2019 database offers comprehensive and comparable data on disease burdens of stomach and esophageal cancer, encompassing numerous useful indicators recorded annually since 1990. In our study, we analysed temporal variations in incidence, mortality rates, and disability-adjusted life-years (DALYs), extracting trends of stomach and esophageal cancers from 1990 to 2019. Furthermore, we projected the cancer burden until 2039 to provide insightful information for stomach and esophageal cancer control in China.

## METHODS

### Data sources

The data of stomach and esophageal cancer data for the 1990–2019 period came from the Global Health Data Exchange (GHDx) query tool (https://ghdx.healthdata.org/gbd-2019). In this study, we obtained data on incidence, mortality, DALYs and related risk factors for stomach and esophageal cancer in China by sex (both sexes, male and female) and age (19 age groups, from <5 to ≥95 years at 5-year intervals) from the GBD Study 2019. All estimates were generated with 95% uncertainty intervals (95% UIs), which were determined based on the 2.5th and 97.5th-ordered percentiles of 1000 draws of the uncertainty distribution. The predicted demographic data for the period 2020–2030 came from the public website (https://ghdx.healthdata.org/record/ihme-data/global-population-forecasts-2017-2100). Socio-demographic index (SDI) varies between 0 and 1, with higher SDI implying better socioeconomic development: based on the SDI, regions are classified into five classes, including low (<0.46), low-middle (0.46–0.60), middle (0.61–0.69), high-middle (0.70–0.81) and high SDI (>0.81). Moreover, regions were also separated into four classes, including high income, upper middle income, lower middle income and low income in term of world bank income. Previous studies described the detailed methodology of GBD study. Then, we retrieved the corresponding population data to predict the ASR of stomach and esophageal cancer among females and males from 2020 to 2030.

The GBD study data followed the guidelines for Accurate and Transparent Health Estimation Reporting for Population Health Research (GATHER).

### Statistical analysis

Estimated average percentage change (EAPC) was computed to describe the trend in ASRs of stomach and esophageal cancer burdens. If the value of EAPC and the lower boundary of 95% CI were bigger than 0, the age-standardised indicators are in an upward trend; if these were less than 0, they are in a decreasing trend; when these were equal to 0, they are a constant trend.

The Bayesian age-period-cohort analysis (BAPC) model with integrated nested Laplace approximation (INLA) was used to predict the incidence and mortality trends of two cancers from 2020 to 2030 in China. All data analyses were performed in Software R (version 4.2.2) and R studio, and the BAPC predictive model used the “nordpred (version 1.1)”, “BAPC (version 0.0.36)” and “INLA (version 22.05.07)” packages. A *P* < 0.05 was considered statistically significant.

## RESULTS

### Burdens of stomach and esophageal cancer in 2019

**Figure 1:**
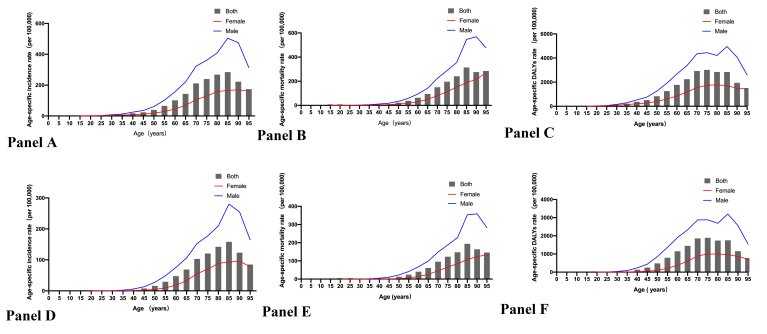
The age-specific incidence, mortality and DALYs rate of stomach and esophageal cancer by age and gender in China in 2019. **Panel A.** The age-specific incidence rate of stomach cancer. **Panel B.** The age-specific mortality rate of stomach cancer. **Panel C.** The age-specific DALYs rate of stomach cancer. **Panel D.** The age-specific incidence rate of esophageal cancer. **Panel E.** The age-specific mortality rate of esophageal cancer. **Panel F.** The age-specific DALYs rate of esophageal cancer. DALYs, disability-adjusted life years, DALY – disability-adjusted life years

There were many newly diagnosed cases of stomach cancer among females and males in 2019. And the male/female ratio of age-specific incidence rate has grown substantially to 2.79 (**Table 1**). Additionally, stomach cancer caused 421 539 related deaths in 2019, with a nearly 71% of male patients (**Table 2**). Furthermore, the DALYs of stomach cancer were as high as 9 824 993 in 2019 (**Table 3**). The age-specific incidence, mortality and DALYs rate among males were all higher than those among females in 2019 (**Figure 1**, panels A, B and C). While the peaks of age-specific incidence and mortality rate among females and males were both in the 85–89 age group, that of age-specific DALYs was in the 75–79 age group.

**Table 1:** The incidence numbers and ASIR of stomach and esophageal cancer in 1990 and 2019 and their temporal trends from 1990 to 2019

	1990		2019		1990-2019
**Characteristics**	**Numbers**	**ASIR per 100 000**	**Numbers**	**ASIR per 100 000**	**EAPC (95% CI)**
**Stomach cancer**					
Both	317 335.29	37.56	612 821.17	30.64	−0.18 (−0.33, 0.01)
Female	109 809.82	25.57	161 489.42	15.80	−0.38 (−0.53, -0.20)
Male	207 525.47	51.07	451 331.75	47.35	−0.07 (−0.29, 0.23)
**Esophageal cancer**					
Both	173 686.86	20.97	278 120.75	13.90	−0.34 (−0.47, −0.13)
Female	58 212.30	13.94	70 197.08	6.83	−0.51 (−0.63, −0.31)
Male	115 474.56	28.70	207 923.67	21.94	−0.24 (−0.42, 0.06)

ASIR – the age-standardised incidence rate, EAPC – estimated average percentage change, CI – confidence interval

**Table 2:** The mortality numbers and ASMR of stomach and esophageal cancer in 1990 and 2019 and their temporal trends from 1990 to 2019

	1990		2019		1990-2019
**Characteristics**	**Numbers**	**ASMR per 100 000**	**Numbers**	**ASMR per 100 000**	**EAPC (95% CI)**
**Stomach cancer**					
Both	305 466.53	37.73	421 539.17	21.72	−0.42 (−0.53, −0.30)
Female	108 410.57	26.17	123 025.46	12.20	−0.53 (−0.63, −0.40)
Male	197 055.96	51.36	298 513.71	33.14	−0.35 (−0.50, −0.16)
**Esophageal cancer**					
Both	176 601.61	22.08	257 315.76	13.15	−0.40 (−0.52, −0.21)
Female	59 516.74	14.69	59 599.82	5.92	−0.60 (−0.69, −0.45)
Male	117 084.87	30.53	197 715.94	21.69	−0.29 (−0.46, −0.01)

ASMR – the age-standardised mortality rate, EAPC – estimated average percentage change, CI – confidence interval

**Table 3:** The DALYs numbers and age standardised DALYs rate of stomach and esophageal cancer in 1990 and 2019 and their temporal trends from 1990 to 2019

	1990		2019		1990-2019
**Characteristics**	**Numbers**	**ASR per 100 000**	**Numbers**	**ASR per 100 000**	**EAPC (95% CI)**
**Stomach cancer**					
Both	8 248 793.39	905.54	9 824 993.42	481.15	−0.47 (−0.57, −0.35)
Female	2 839 182.00	619.82	2 689 002.10	260.97	−0.58 (−0.67, −0.44)
Male	5 409 611.38	1,205.79	7 135 991.33	718.79	−0.40 (−0.55, −0.21)
**Esophageal cancer**					
Both	4 494 070.33	506.98	5 759 997.30	277.50	−0.45 (−0.57, −0.22)
Female	1 366 551.29	311.63	1 137 699.24	172.06	−0.65 (−0.73, −0.49)
Male	3 127 519.04	707.12	4 622 298.06	458.55	−0.35 (−0.52, −0.04)

DALYs – disability-adjusted life years, ASR – age-standardised DALYs rate, EAPC – estimated average percentage change, CI – confidence interval

The number of newly diagnosed cases of esophageal cancer among males was close to 278 121, accounting for 74.76% of all new patients in 2019 (**Table 1**). In addition, there were many cancer-related deaths in 2019, almost close to the number of new cases (**Table 2**). While the DALYs of esophageal cancer among females were 1 137 699, those among males were 4 622 298 (**Table 3**). Interestingly, the age-specific incidence, mortality and DALYs rate among females and males, as well as the peaks among females and males in esophageal cancer, were nearly identical to those in stomach cancer (**Figure 1**, panels D, E and F).

### Trends of stomach and esophageal cancer from 1990 to 2019

**Figure 2:**
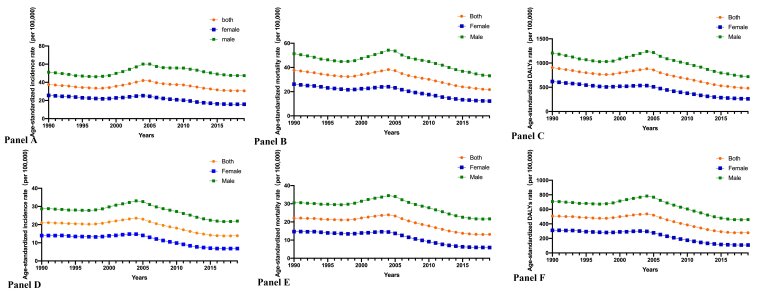
The age-standardised incidence, mortality and DALYs rate of stomach and esophageal cancer in 1990-2019. **Panel A.** The ASIR of stomach cancer. **Panel B.** The ASMR of stomach cancer. **Panel C.** The age-standardised DALYs rate of stomach cancer. **Panel D.** The ASIR of esophageal cancer. **Panel E.** The ASMR of esophageal cancer. **Panel F.** The age-standardised DALYs rate of esophageal cancer. ASIR – the age-standardised incidence rate, ASMR – the age-standardised mortality rate, DALYs – disability-adjusted life years

The ASIR of stomach cancer in China decreased from 1990 to 2019 (EAPC = −0.18; 95% CI = −0.33 to 0.01), as was shown in **Table 1**. While the ASIR for males during the period manifested an overall stable trend (EAPC = −0.07; 95% CI = −0.29 to 0.23), that for females showed a nearly continuous downward trend (EAPC = −0.38; 95% CI = −0.53 to −0.20) (**Table 1**; **Figure 2**, panel A). Moreover, the ASMR of stomach cancer in China had a generally significant decline during the period (EAPC = −0.42; 95% CI = −0.53 to −0.30). And the ASMR decreased in both females and males, with a more pronounced downward trend in females (**Table 2**; **Figure 2**, panel B). During this period, the age-standardised DALYs dropped a lot, which showed a more conspicuous decline in females (EAPC = −0.58; 95% CI = −0.67 to −0.44) (**Table 3**; **Figure 2**, panel C).

There was a lower ASIR of esophageal cancer in 2019 than in 1990 (EAPC = −0.34; 95% CI = −0.47 to −0.13). The ASIR among females (EAPC = −0.51; 95% CI = −0.63 to −0.31) exhibited a more obvious downward trend than that of males (EAPC = −0.24; 95% CI = −0.42 to 0.06) (**Table 1**; **Figure 2**, panel D). From 1990 to 2019, the ASMR of esophageal cancer in China exhibited an overall decline (EAPC = −0.40; 95% CI = −0.52 to −0.21). Compared to a substantial decline in ASMR among females from 1990 to 2019 (EAPC = −0.60; 95% CI = −0.69 to −0.45), the ASMR of males showed a relatively smoother descent during the period (EAPC = −0.29; 95% CI = −0.46 to −0.01) (**Table 2**; **Figure 2**, panel E). During this period, the age-standardised DALYs also decreased a lot (EAPC = −0.45; 95% CI = −0.57 to −0.22). While the age-standardised DALYs for males during the period manifested an overall smooth downward trend (EAPC = −0.35; 95% CI = −0.52 to −0.04), those for females showed a drastic downward trend (EAPC = −0.65; 95% CI = −0.73 to −0.49) (**Table 3**; **Figure 2**, panel F).

### Risk factors for stomach and esophageal cancer

**Figure 3:**
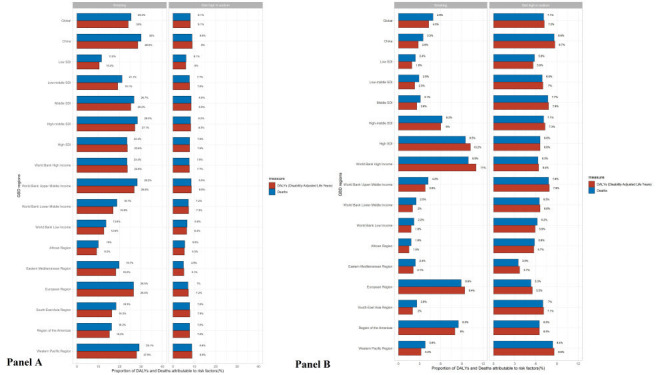
Proportion of stomach cancer DALYs and deaths attributable to risk factors, for global, China and 15 regions in 2019 by sex. **Panel A.** Stomach cancer in females. **Panel B.** Stomach cancer in males. DALYs – disability-adjusted life years

We analysed 87 risk factors from the GHDx website and identified two primary risk factors for stomach cancer: smoking and a diet high in sodium. In 1990, both were associated with a higher disease burden of stomach cancer (Figures S1 and S2 in the **Online Supplementary Document**). While smoking has a greater effect on DALYs and death rate in males, diet high in sodium played a bigger role in these in females. In 2019, the influence of smoking on DALYs and death rates in China increased slightly, while other results remained relatively stable (**Figures 3**, panels A and B). We also observed that high socioeconomic development index (SDI), high-income countries, and the European region were more affected by smoking in males, while low SDI, low-income countries and the East Mediterranean region were more affected by a high-sodium diet in females. Furthermore, the proportion of risk factors attributable to stomach cancer in China was closer to that of upper-middle-income countries than high-income countries.

**Figure 4:**
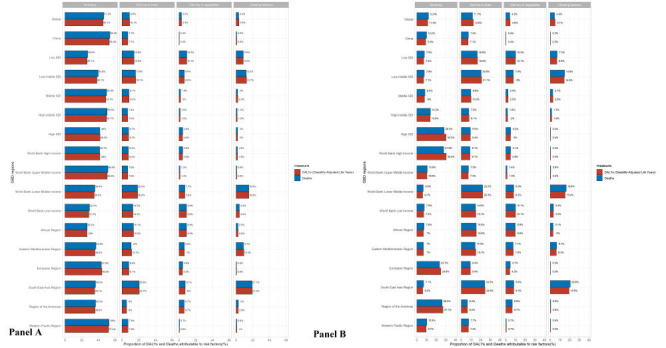
Proportion of esophageal cancer DALYs and deaths attributable to risk factors, for global, China and 15 regions in 2019 by sex. **Panel A.** Esophageal cancer in females. **Panel B.** Esophageal cancer in males. DALYs – disability-adjusted life years

The four primary risk factors for esophageal cancer were also analysed and identified from the 87 risk factors. Globally, smoking, a diet low in fruits or vegetables, and chewing tobacco were associated with a higher disease burden of esophageal cancer. In comparison to the attributable risks in 1990 (Figures S3 and S4 in the **Online Supplementary Document**), the impact of these risk factors in 2019 slightly decreased. However, smoking remained the most significant risk factor in males, while a diet low in fruits was the most significant risk factor in females. Interestingly, a diet low in vegetables and chewing tobacco had minimal impact on esophageal cancer risk. We also examined the risk factors in different Global Burden of Disease (GBD) regions and found that the proportion of risk factors in China was similar to that of high-middle SDI and upper-middle-income countries and was nearly identical to that in the Western Pacific region (**Figure 4**, panels A and B).

### Predictions of stomach and esophageal cancer from 2020 to 2030

**Figure 5:**
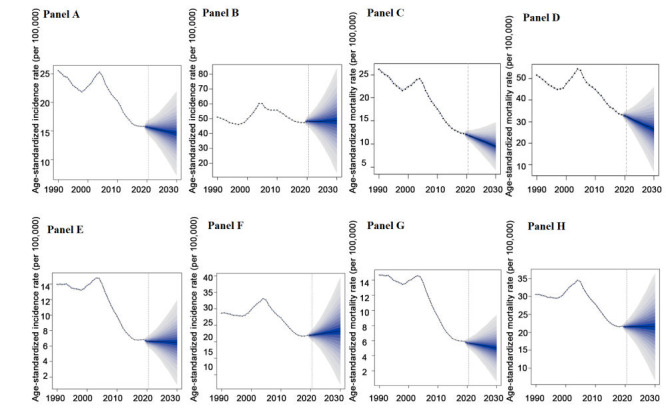
The change trends of the cancer-related disease burden by sex from 1990 to 2030 in China. **Panel A.** The ASIR of stomach cancer among females. **Panel B.** The ASIR of stomach cancer among males. **Panel C.** The ASMR of stomach cancer among females. **Panel D.** The ASMR of stomach cancer among females. **Panel E.** The ASIR of esophageal cancer among females. **Panel F.** The ASIR of esophageal cancer among males. **Panel G.** The ASMR of esophageal cancer among females. **Panel H.** The ASMR of esophageal cancer among males. ASIR – the age-standardised incidence rate, ASMR – the age-standardised mortality rate

Utilising Bayesian age-period-cohort (BAPC) models, we predict the ASIR of stomach and esophageal cancer in different sex from 2020 to 2030. We found that the ASIR of stomach cancer in females will experience a continuous decline from 2020 to 2030. However, the ASIR for males is predicted to show a slight increase during the same period (**Figure 5**, panels A and B). Additionally, we forecast that the ASMR of stomach cancer in both males and females will significantly decrease over this timeframe, consistent with the downward trend observed from 2010 to 2019 (**Figure 5**, panels C and D). What’s more, we predict that the ASIR and ASMR of esophageal cancer in females will both exhibit a gradual and steady decrease from 2020 to 2030 (**Figure 5**, panels E and F). In contrast, the ASIR and ASMR of esophageal cancer in males are expected to display stable and smooth trends during this period, which markedly differs from the substantial decline observed from 2010 to 2019 (**Figure 5**, panels G and H).

## DISCUSSION

The high morbidity and mortality of stomach and esophageal cancer globally demand increased attention to these cancers and efforts to reduce their severe damage [22]. According to the latest reports, new cases and death cases of stomach and esophageal cancer in China account for nearly half worldwide every year [23]. Most of these cancers are closely related to eating habits, such as pickled and leftover foods[24–26]. These foods, which often appear on the Chinese table, contain a large amount of highly carcinogenic nitrite. Thus, it is crucial to strongly advocate for the Chinese population to eat less pickles and moldy foods and more fresh foods.

Most studies have reported that the risk of stomach and esophageal cancer is nearly two times higher in males than in females [22]. In our study, we found that the male-to-female ratio of new cases of both cancers in China was more than two times in 2019, particularly for esophageal cancer, which was over four times higher. The increased risk in males may be due to their higher likelihood of exposure to alcohol and tobacco [26]. Additionally, we observed that patients of both sexes older than 75 years were more likely to develop both cancers. This could possibly be attributed to the elderly having too low immunity to resist the development of both cancers [22]. Our study identified male sex, older age, smoking, and alcohol consumption as factors associated with heavier disease burdens for both cancers, supporting previously suggested causes. We also found that obesity and gastroesophageal reflux disease were important risk factors for both cancers. Prior studies have concluded that patients with high BMI or gastroesophageal reflux disease are more likely to develop stomach and esophageal cancer, emphasising the need for public policies to control these risk factors [27–29]. Therefore, there need be more rational public policies to control these risk factors.

From 1990 to 2019, the ASRs of stomach and esophageal cancer all had a gradual overall downward trend in China. However, our BAPC predictive model showed a stable or upward trend of ASRs among male patients, especially for ASIRs of both cancers. It is worth mentioning that the number of new cases of both cancers first decreased and then increased from 1990 to 2005. The national cancer prevention and treatment publicity week initiated by the Chinese Anti-cancer Association in 1995 increased public awareness of cancers, leading to early examination and treatment, which may have contributed to a temporary decline. However, with the establishment of the National Central Cancer Registry in 2002, the quality of cancer data in China improved dramatically, resulting in a new rise in new cases of both cancers. The ASIR, ASMR and age-standardised DALYs of both cancers among females and males showed significant downward trends in China from 2005 to 2019, largely due to overall improvements in Chinese health care. They should have continued to decrease after 2019.

Addressing the substantial burden of stomach and esophageal cancer, which is expected to increase in the coming years, requires cost-effective strategies. First, it is essential to raise national awareness by spreading knowledge about cancer prevention and treatment. Second, early examinations and treatment are effective actions to greatly improve the survival rate and quality of life. Third, increased investment in cancer research to improve treatment is necessary and urgently needed.

However, there are some limitations in this study. First, our study is a secondary data analysis from the GBD study, which has unavoidable shortcomings such as data quality assurance. Second, there are many risk factors related to these cancers, but we only selected a few factors for further discussion. Third, the predictive model roughly described the future trend, but can’t provide accurate forecasts. Beyond these limitations, we need to translate the results of our study into practical actions, such as developing public policy and informing subsequent research.

## CONCLUSIONS

Stomach and esophageal cancer have imposed substantial disease burdens in China. Nevertheless, the incidence and mortality rates have significantly decreased due to the implementation of national policies and advancements in cancer diagnosis and treatment. The forecast model also indicated that overall condition will improve in the next decade. Maintaining healthy lifestyle habits, such as reducing tobacco and alcohol consumption, avoiding moldy foods, and increasing the intake of fresh fruits and vegetables, can lower the risk of stomach and esophageal cancer. Consequently, it is of great importance for China to develop and implement policies aimed at reducing these risk factors.

## Additional material


Online Supplementary Document
