# Supplementary material for: Burdens of stomach and esophageal cancer from 1990 to 2019 and projection to 2030 in China: Findings from the 2019 Global Burden of Disease Study
Source: J Glob Health. 2024 Jan 5;14:04025. doi: 10.7189/jogh.14.04025 (PMC10769142; doi:10.7189/jogh.14.04025)
Supplement: Online Supplementary Document [file jogh-14-04025-s001.pdf]

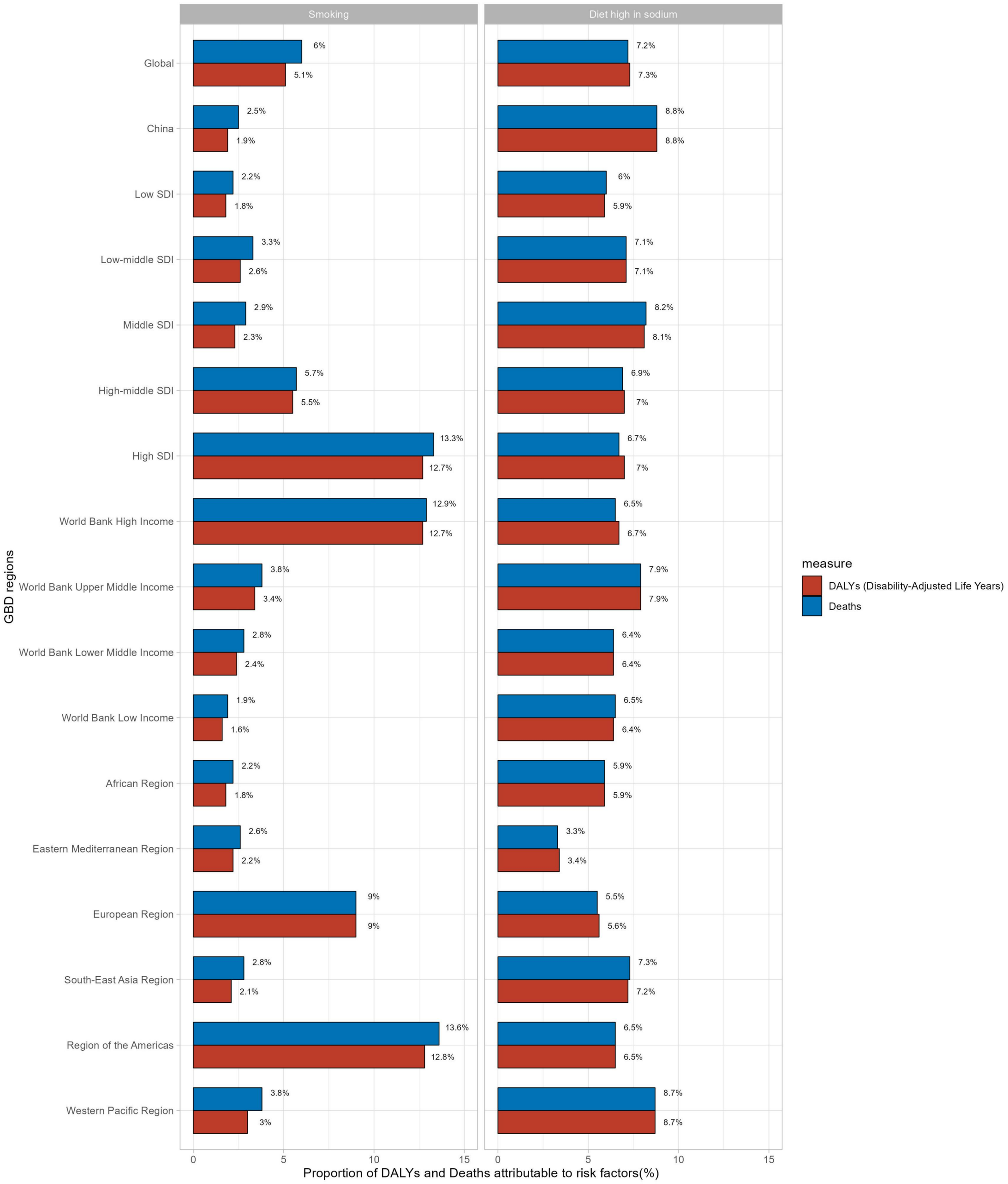

Supplementary Figure 1: Proportion of DALYs and Deaths attributable to risk factors among female stomach cacner patients.

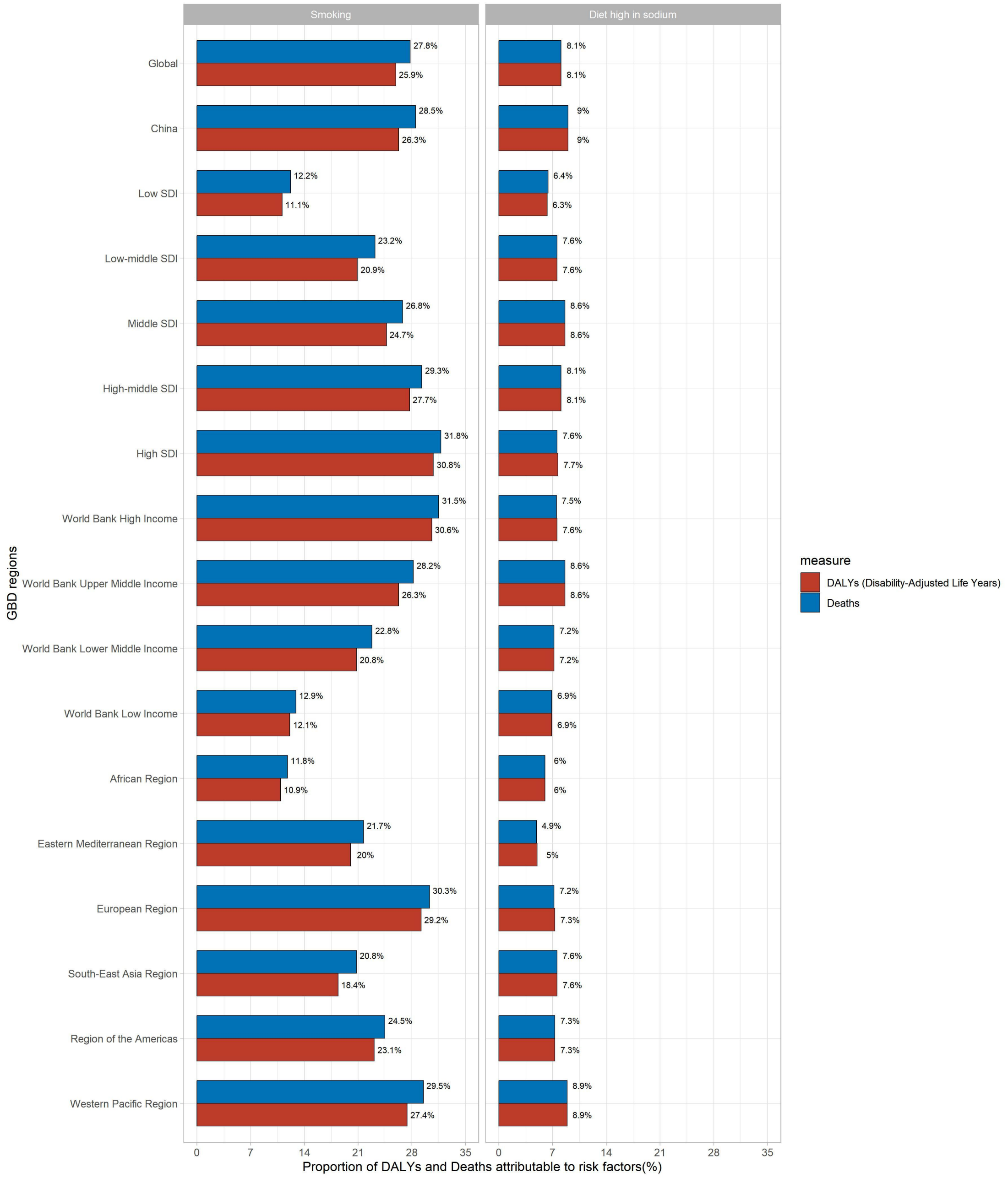

Supplementary Figure 2: Proportion of DALYs and Deaths attributable to risk factors among male stomach cacner patients.

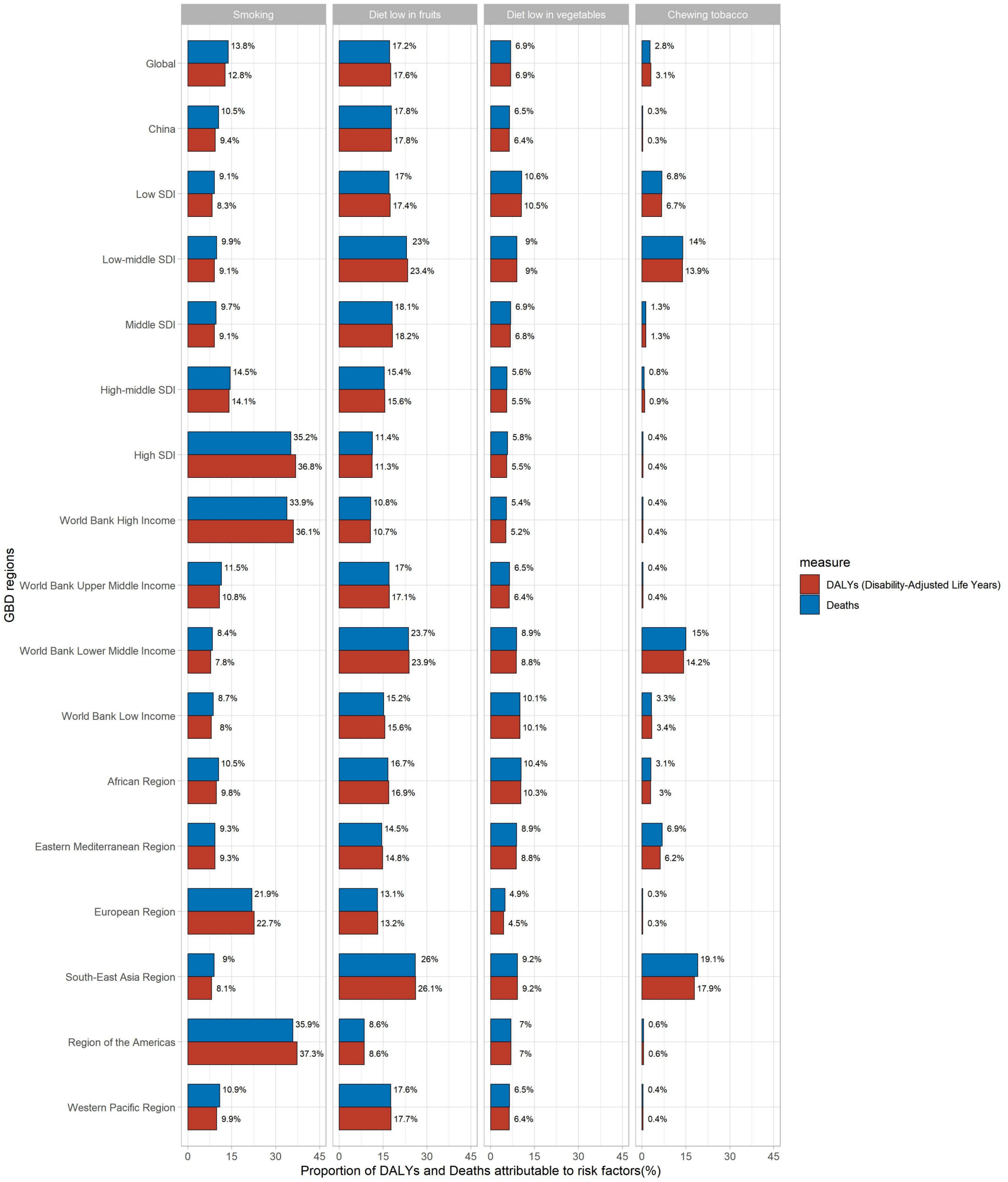

Supplementary Figure 3: Proportion of DALYs and Deaths attributable to risk factors among female esophageal cancer patients.

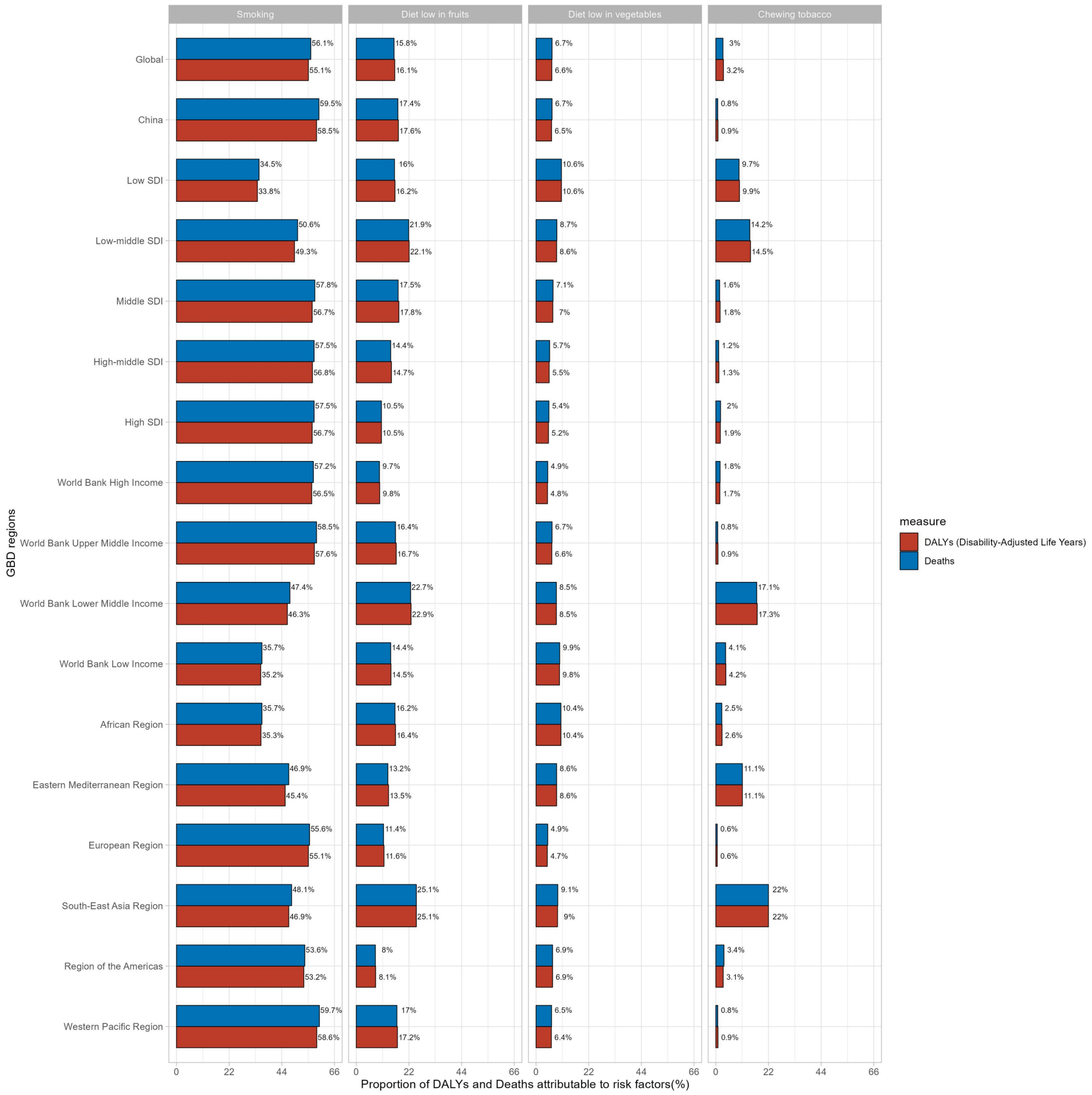

Supplementary Figure 4: Proportion of DALYs and Deaths attributable to risk factors among male esophageal cancer patients.
